# Crohn's disease or histoplasmosis? A case of severe disseminated histoplasmosis mimicking Crohn's disease and literature review

**DOI:** 10.1016/j.mmcr.2020.08.003

**Published:** 2020-08-28

**Authors:** Ahmed Ahmed, Nora Homsi, Rajendra Kapila

**Affiliations:** a Division of Medicine, Rutgers New Jersey Medical School, Newark, NJ, USA; b Division of Infectious Disease, Rutgers New Jersey Medical School, Newark, NJ, USA

**Keywords:** Histoplasmosis, Gastrointestinal bleeding, Inflammatory bowel disease, Immunosuppression

## Abstract

Disseminated histoplasmosis (DH) often mimics other diseases, leading to misdiagnosis and delays in treatment. We present a patient who developed DH after treatment with immunosuppressants for an initial diagnosis of inflammatory bowel disease (IBD). Upon diagnosing her with DH, liposomal amphotericin B was started, and she eventually recovered after a prolonged hospitalization. Intrabdominal histoplasmosis has many similarities with IBD. Treatment with immunosuppressants in undiagnosed histoplasmosis can lead to dissemination with potentially catastrophic results.

## 1 Introduction

Histoplasmosis, a mycotic infection caused by strains of Histoplasma, is known to affect people worldwide; however, it is mostly found in North and Central America [1]. Known endemic regions include the Caribbean, parts of Ecuador, Venezuela, and the Ohio Mississippi River valley region [2,3]. Found in the environment, this fungus tends to inhabit soil containing large amounts of bird or bat droppings and is acquired via inhalation of airborne microconidia [[1], [2], [3]]. The clinical presentation is variable, ranging anywhere from asymptomatic to disseminated disease, can involve any organ, and is strongly influenced by various factors such as age, host immunity, and intensity of exposure [1,4]. In disseminated disease, about 70% of patients are found to have gastrointestinal (GI) involvement at autopsy, yet only about 10% of cases will demonstrate GI clinical manifestations [5,6]. Lesions in the GI tract can appear as ulcerations and occur anywhere, with the colon and ileum being the most affected [5,7]. Histologically, infiltration of macrophages laden with yeast, perivasculitis with necrosis, and non-caseating granulomas can be seen [5,8]. Due to similarities with IBD, histoplasmosis may be misdiagnosed as Crohn's disease or ulcerative colitis [5].

We hereby present a 38-year-old Ecuadorian woman who presented to an outside hospital (OSH) with abdominal pain and a GI bleed and was diagnosed with presumed Crohn's disease and treated with immunosuppressants. Despite adherence to treatment, her symptoms continued to worsen, requiring rehospitalization at the OSH and multiple abdominal surgeries. Due to post-operative complications and her critical condition, she was subsequently transferred to our facility for further management and required an interdisciplinary approach to ultimately diagnose and treat her for severe DH. Such a case and literature review enhances the medical recognition of the vast presentation of histoplasmosis and illustrates the importance of ruling out endemic infections such as histoplasmosis prior to initiating immunosuppressive therapies.

## 2 Case presentation

A 38-year-old Ecuadorian woman with a past medical history of treated hepatitis C and a new presumed diagnosis of Crohn's disease presented as a transfer from an OSH in septic shock and respiratory failure due to complications after multiple abdominal surgeries for an uncontrolled GI bleed.

Approximately three months prior to her transfer, she presented to an OSH with severe upper abdominal pain and bloody diarrhea for several weeks, prompting an endoscopic evaluation. She was diagnosed with Crohn's Disease and was discharged on mesalamine (1 g four times a day). Since her discharge, she was adherent with her medication but continued to have abdominal pain, nausea, and bloody diarrhea. She was readmitted one month later for a presumed Crohn's flare and then discharged on oral prednisone (40 mg daily for 2 weeks, followed by a slow taper) and mesalamine (1g four times a day). One month prior to her transfer, she presented again to the OSH for worsening emesis, bloody diarrhea, subjective fevers, and abdominal pain. She was noted to be septic with a maximum temperature of 103 °F (39.4 °C) and acutely anemic from an ongoing GI bleed that required multiple transfusions. Results of the computerized tomography (CT) scan were concerning for an intrabdominal abscess and empiric intravenous (IV) antibiotics (meropenem 1 g every 8 hours and vancomycin 1 g every 12 hours) were started. Endoscopic treatment of the GI bleed failed due to inability to localize it; thus, she underwent a total colectomy with a Brooke ileostomy. Due to ongoing abdominal pain, she underwent repeat abdominal CT, findings of which were concerning for a small bowel laceration. She subsequently underwent interventional radiologic (IR) drain placement and was continued on IV meropenem and vancomycin. Shortly thereafter, she developed septic shock and acute respiratory failure and was subsequently transferred to our institution, intubated and on vasopressors.

Upon arrival to our hospital (day 0), she was tachycardic and febrile to 102 °F. Her laboratory findings were notable for leukocytosis of 12.4 × 10^3^/μL, thrombocytopenia of 40 × 10^3^/μL, anemia with hemoglobin of 10.9 g/dL, INR of 2.0, total bilirubin of 2.6 mg/dL, direct bilirubin of 1.2 mg/dL, and aspartate aminotransferase (AST) of 56 U/L. A repeat CT scan revealed bilateral pleural effusions and consolidations of the lungs, ascites, peritonitis with a loculated fluid and air collection concerning for an abscess, small adrenal lesions, bilateral hypoattenuating lesions within the kidney concerning for abscesses, and pneumoperitoneum. As a result, the patient underwent an exploratory laparotomy on day 1 with washout and enterotomy repair and right chest tube placement. Peritoneal cultures from the washout as well as blood cultures grew ESBL *E. coli.* From days 2–3, she was noted to have continuous blood loss in the ileostomy bag with worsening anemia nonresponsive to blood transfusions in the setting of disseminated intravascular coagulation (DIC). Due to the patient's borderline hemodynamic status and rapid blood loss, she was not deemed an endoscopic candidate and underwent an abdominal angiogram by IR on day 4. She was noted to have a brisk hemorrhage from a branch of the superior mesenteric artery (SMA) which required embolization with coils and gelfoam. Post embolization, her GI blood loss improved; however, subsequent serum analysis from days 4–5 continued to demonstrate down trending hemoglobin and platelet count and up trending liver function tests (LFTs). An ultrasound showed a thickened gallbladder with sludge but was devoid of calculi and possible common bile duct (CBD) dilation, prompting a magnetic resonance cholangiopancreatography (MRCP) of the abdomen which only showed mild pericholecystic fluid and a thickened gallbladder.

**Fig. 1:**
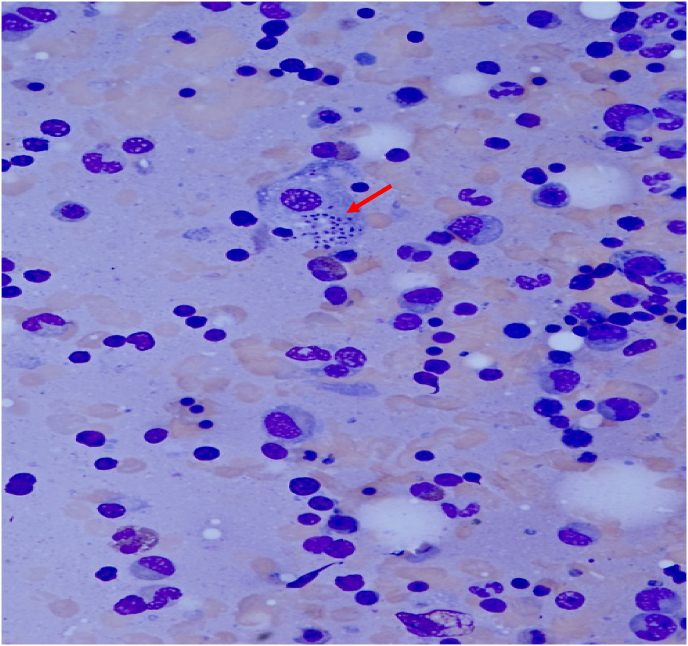
Romanowsky–Giemsa staining (40X magnification) of a sample of the resected colon demonstrating *Histoplasma capsulatum* (solid red arrow) within a macrophage.

**Fig. 2:**
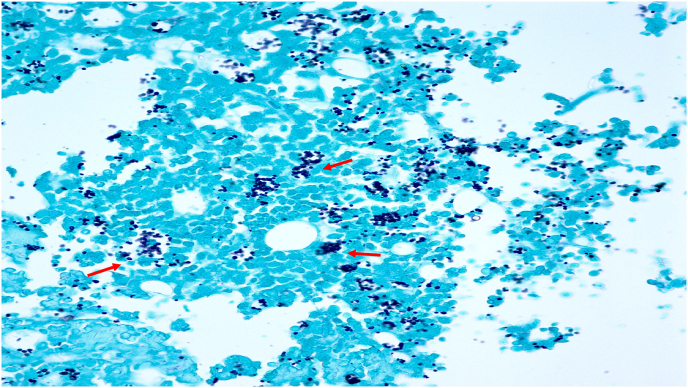
Grocott methenamine silver (GMS) stain (20x magnification) of the resected colon demonstrating an abundance of *Histoplasma capsulatum* (solid red arrows) within cells around several vascular lumina, consistent with severe disseminated histoplasmosis.

**Fig. 3:**
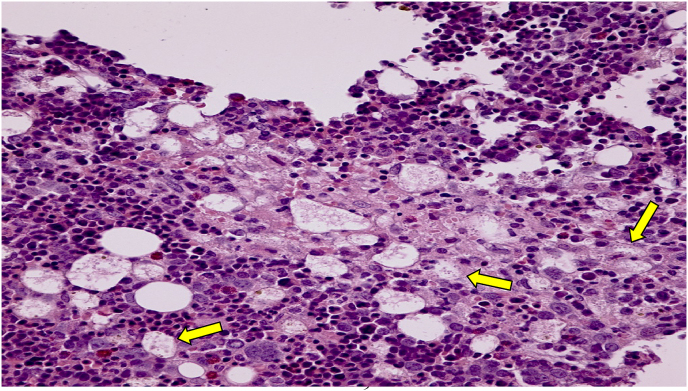
A Hematoxylin and eosin (H&E) stain (20X magnification) of the resected colon showing small (1–2mm) organisms consistent with *Histoplasma capsulatum* in histiocytes (solid yellow arrows).

Pathology samples of the resected colon from the OSH were obtained on day 5 and demonstrated histoplasma-laden macrophages present in the vascular lumina throughout the colon consistent with severe histoplasma associated colitis [Fig. 1, Fig. 2, Fig. 3]. Biopsies obtained during her initial esophagogastroduodenoscopy (EGD) and colonoscopy demonstrated diffuse inflammation with several noncaseating granulomata but were not stained for fungal entities. Due to the evidence of severe DH, the patient was started on IV liposomal amphotericin B (3 mg/kg daily) on day 5. DH was further confirmed with positive serum and urine histoplasma antigens and growth of histoplasma in blood cultures. Ophthalmologic evaluation was negative for any ocular involvement. Further workup was negative for other infections including human immunodeficiency virus (HIV), human T-lymphotropic virus (HTLV), Epstein-Barr virus (EBV), cytomegalovirus (CMV), hepatitis B, hepatitis A, *Clostridioides difficile*, and interferon gamma release assay.

By day 11, the patient was later found to have fecal matter oozing from her abdominal surgical sites and had another exploratory laparotomy with abdominal washout and drain placement. After the surgery, she was monitored closely and was assessed by physical therapy and nutritional services for her malnutrition and cachexia. Following these interventions, the patient improved clinically and was successfully extubated on day 19. A repeat CT of the abdomen showed resolution of the renal and peritoneal abscesses and the pleural effusions. Due to the severity of the histoplasma infection in conjunction with the multiple abdominal surgeries she underwent, and the resulting malabsorption, she was continued on IV liposomal amphotericin B for a total duration of three months and was then transitioned to oral itraconazole (200 mg three times a day for three days followed by 200 mg twice a day) to complete one year of antifungal therapy. She was eventually discharged to a subacute rehabilitation facility with close outpatient follow up and is now back to her baseline functional status.

## 3 Discussion

Although there are various strains of Histoplasma, *H. capsulatum* is the most common and is primarily found in soil contaminated with bird excreta or bat guano [1,2]. *H. capsulatum* has a propensity to thrive in regions such as Central America since soil analyses demonstrate the organism's preference to live between a pH of 5–10 or at temperatures greater than 40 °C [4].

Though the clinical spectrum of histoplasmosis is vast, most infections are asymptomatic or self-limited and is strongly influenced by factors such as age, immunological status of the host, and amount of inoculum [1,4]. One in 2000 patients can develop acute pulmonary infections or severe disseminated and progressive presentations of the disease [1,7]. Disseminated disease usually occurs in individuals who are immunocompromised; particularly in HIV infection, hematologic malignancies, and treatment with corticosteroids or tumor necrosis factor antagonists [2]. Our patient's presentation suggests that she had GI involvement which then disseminated after initiation of immunosuppressants.

Depending on the organ involvement, the symptoms of DH can be non-specific and include fever, weight loss, and malaise. DH can involve any organ system, with a propensity towards the reticuloendothelial system, skin, GI tract, and adrenal gland [1]. Infection occurs when histoplasma microconidia are inhaled into the lungs and transition into the yeast form. In an immunocompetent host, neutrophils, macrophages, lymphocytes and natural killer cells are involved in the inflammatory response [9]. It is thought that hematogenous dissemination occurs during the acute infection prior to the development of cellular immunity [5,9]. Macrophages are the key cells involved in the spreading of the organism via the lymphatic and blood vessels. Once infected, T Cell immunity is the predominant defense mechanism, with cytokines such as interleukin (IL)-12 and interferon (IFN)-gamma activating macrophages acting as important moderators [10,11]. This defense mechanism is typically sufficient to halt disease progression in an immunocompetent host leading to mostly subclinical histoplasmosis presentations. However, as eluded to before, immunocompromised patients have impaired defenses which lead to an increased risk of developing fatal disseminated disease [2]. A retrospective study of 61 patients with DH reported a mortality rate of 31% in immunocompromised patients and 17% in immunocompetent patients [12].

Diagnosing histoplasmosis requires a high index of suspicion and knowledge of risk factors and endemic regions. Laboratory and radiographic findings vary and include elevated LFTs, pancytopenia, lymphadenopathy (LAD), or diffuse interstitial or reticulonodular pulmonary infiltrates [13].

Blood cultures and antigen testing are key studies that should be performed in all suspected histoplasmosis cases. Both serum and urine antigen studies can be done, with a preference of performing both, as urine studies can sometimes be falsely negative [14]. Moreover, in immunocompetent patients, histoplasma antigen was detected in the urine in approximately 75% of cases while it was detected in 95% of immunocompromised patients with disseminated disease [14]. Although there are immunodiffusion and complement fixation tests for antibodies against histoplasma, they are often falsely negative in acutely ill or immunosuppressed patients, with one study showing only 38% sensitivity in solid organ transplant patients [15].

Histopathologically, different tissue responses have been described in DH with histiocytosis or macrophage infiltration being a common finding. Other findings include perivasculitis with necrosis, which is sometimes found in severe infections [5]. More pertinent to this case is the sarcoid-like, non-caseating granuloma formation that can occur with histoplasma infection. Like other granuloma-inducing infections, granuloma formation in histoplasmosis is a type of defense mechanism used to contain the organism and prevent systemic dissemination [8]. A histoplasma-induced delayed-type hypersensitivity response with IFN-γ-producing CD4^+^ T cells and activated macrophages producing TNF-α are key factors to produce the diffuse sarcoid-type granuloma formation [8].

In DH, approximately 70% of patients can have GI involvement but approximately 10% of cases will have clinical manifestations of GI involvement, illustrating a rare aspect of this case [5,6]. Morphologically, histoplasmosis lesions of the GI tract can appear as ulcerations or polypoid masses and often involves the colon or ileum but may occur anywhere along the GI tract [5,7]. Histologically, the GI tract will often demonstrate histoplasma-invaded macrophages in the submucosa and lamina propria [5,6]. Due to similarities, histoplasmosis is sometimes misdiagnosed as IBD, as was the case in our patient [6]. Thus, it is imperative to exclude histoplasmosis in cases where IBD is suspected prior to initiating immunosuppressive therapy, especially in patients with risk factors or exposure to endemic areas [6].

Treatment options for histoplasmosis depend on the severity of the disease and whether the central nervous system (CNS) is involved. In patients with CNS involvement or with severe disseminated disease, a lipid formulation of amphotericin B is indicated initially with a transition to itraconazole. Itraconazole is not used initially since serum levels takes several weeks to reach a steady state, making it less effective in the acute phase of treatment for severe disease [14]. Transitioning from amphotericin B to itraconazole is typically initiated once the patient becomes afebrile, is hemodynamically stable, and can tolerate oral medications. In mild to moderate disease, itraconazole is usually the treatment of choice [16]. Regardless of disease severity, the total duration of treatment should be at least one year to reduce the risk of relapse [7,16].

In the present case, the patient was from Ecuador and presented with GI symptoms (GI bleeding, abdominal pain). She was started on mesalamine and then subsequently corticosteroids for presumed Crohn's disease. DH in the GI tract can mimic endoscopic findings of IBD; thus, it is likely that the patient's initial presentation that prompted endoscopic examination was the clinical manifestation of histoplasmosis. This is further evident by the worsening of symptoms upon initiation of immunosuppressants that likely precipitated further dissemination of the infection. Moreover, the specimen slides from the total colectomy showed diffuse, severe histoplasma associated colitis with organisms evident throughout the colon as well as sarcoid-like, noncaseating granulomas. Such a histological presentation further demonstrates the likelihood of misdiagnosing histoplasmosis as IBD since Crohn's disease is known to have a similar histological appearance on biopsies, specifically non-caseating granulomas which is found in up to 30% of patients [[17], [18], [19]]. Unfortunately, the endoscopic biopsy slides were never stained with any fungal stains, which led to the misdiagnosis of Crohn's disease and delay in appropriate treatment.

This case demonstrates the need for clinicians to develop and adhere to strict protocols prior to initiating immunosuppressive treatments. These protocols should include infection screening that is guided by the patient's travel and exposure history. Had such protocols been in place, our patient would have been screened for several infections, including histoplasmosis which is endemic to Ecuador, prior to starting immunosuppressants.

## Funding source

There are none.

## Consent

Written informed consent was obtained from the patient or legal guardian(s) for publication of this case report and accompanying images. A copy of the written consent is available for review by the Editor-in-Chief of this journal on request.

## Declaration of competing interest

There are no conflicts of interest to declare for any authors.
